# Supplementary material for: Effects of different exercise training programs on the functional performance in fibrosing interstitial lung diseases: A randomized trial
Source: PLoS One. 2022 May 26;17(5):e0268589. doi: 10.1371/journal.pone.0268589 (PMC9135240; doi:10.1371/journal.pone.0268589)

Supplemental material

Methods

mMRC dyspnea scale was scored between 0 to 4 whereas 0: breathlessness only on strenuous exercise, 1: breathlessness when hurrying on ground level or walking up a slight hill, 2: walking slower than people in the same age on ground level due to breathlessness or stopping for breath when walking at their own pace on the ground level, 3: stop for breath on ground level after walking about 100 meters or after few minutes, and 4: too breathless when change their clothes or breathless to leave the house.

Results

Table S1: Baseline CPET variables (at 0 watts of workload) before ET among the studied 3 groups

| **Variable** | **Control group**  **(n=11)** | **LL group**  **(n=10)** | **ULB group**  **(n=10)** | **Sig.**  **(*p* value)** |
| --- | --- | --- | --- | --- |
| VT (L) | 0.68 ± 0.14 | 0.61 ± 0.16 | 0.55 ± 0.14 | 0.178 |
| VE (L/min) | 18.58 ± 4.03 | 16.19 ± 4.95 | 14.88 ± 4.64 | 0.225 |
| VE% | 25.7 (16.6 – 44.8) | 25.7 (18.7 – 28.1) | 24.1 (19.5 – 29.2) | 0.872 |
| VO_2_ (L/min) | 0.45 (0.40 – 0.52) | 0.40 (0.36 – 0.47) | 0.34 (0.24 – 0.40) | 0.008* |
| VO_2_% | 30.8 (23.8 – 34.1) | 23.1 (21.2 – 25.4) | 17.6 (13.0 – 25.5) | 0.017* |
| VO_2_/kg (ml/kg/min) | 7.22 ± 1.79 | 6.90 ± 2.28 | 4.50 ± 2.12 | 0.015* |
| VO_2_/kg% | 33.6 (21.5 – 40.0) | 27.2 (19.8 – 34.5) | 16.4 (15.4 – 32.0) | 0.997 |
| VCO_2_ (L/min) | 0.29 ± 0.13 | 0.25 ± 0.07 | 0.22 ± 0.06 | 0.311 |
| Respiratory rate (br/min) | 27.40 (23.26 – 33.02) | 25.98 (23.62 – 27.78) | 26.63 (22.39 – 28.57) | 0.931 |
| HR (b/min) | 91.43 ± 12.92 | 101.38 ± 17.06 | 110.13 ± 15.18 | 0.084 |
| HR% | 57.9 (55.0 – 60.3) | 60.3 (49.8 – 65.7) | 60.6 (58.6 – 67.9) | 0.680 |
| VO_2_/HR (ml/beat) | 5.6 ± 2.40 | 4.29 ± 1.66 | 2.7 ± 0.95 | 0.015* |
| VO_2_/HR% | 56.5 (48.6 – 108.5) | 62.4 (44.7 – 91.2) | 59.5 (35.3 – 127.5) | 0.997 |
| PETCO_2_ (mmHg) | 23.89 ± 4.94 | 23.60 ± 4.43 | 23.60 ± 2.80 | 0.985 |
| PETO_2_ (mmHg) | 113.0 (108.75 – 116.5) | 109.5 (101.0 – 114.0) | 118.0 (114.0 – 119.0) | 0.083 |
| SpO_2_ (%) | 91.5 ± 3.72 | 93.3 ± 2.79 | 92.3 ± 2.11 | 0.405 |
| Breathing Reserve (%) | 81.11 ± 5.58 | 83.70 ± 7.56 | 86.40 ± 4.40 | 0.179 |
| VD/VT ratio | 0.35 (0.23 – 0.36) | 0.24 (0.18 – 0.28) | 0.21 (0.16 – 0.26) | 0.065 |

Table S2: Comparison between CPET at baseline (at 0 watts of workload) before and after ET among group LL and ULB

| **Variable** | **LL group before ET**  **(n=10)** | **LL group after ET**  **(n=10)** | **Sig.**  **(*p*) ^#^** | **ULB group before ET**  **(n=10)** | **ULB group after ET**  **(n=10)** | **Sig.**  **(*p*) ^#^** | **Sig.**  **(*p*)** ^$^ |
| --- | --- | --- | --- | --- | --- | --- | --- |
| VT (L) | 0.61 ± 0.16 | 0.70 ± 0.22 | 0.107 | 0.55 ± 0.14 | 0.61 ± 0.17 | 0.097 | 0.277 |
| VE (l/min) | 16.19 ± 4.95 | 16.82 ± 6.33 | 0.645 | 14.88 ± 4.64 | 14.54 ± 2.55 | 0.743 | 0.303 |
| VE% | 25.7 (18.7 – 28.1) | 22.6 (17.7 – 29.8) | 0.959 | 23.1 (18.6 – 27.1) | 24.1 (19.5 – 29.2) | 0.386 | 0.762 |
| VO_2_ (L/min) | 0.40 (0.36 – 0.47) | 0.37 (0.31 - 0.48) | 0.508 | 0.34 (0.24 – 0.40) | 0.35 (0.30 - 0.47) | 0.036* | 0.436 |
| VO_2_% | 23.1 (21.2 – 25.4) | 20.7 (19.1 – 23.6) | 0.508 | 17.6 (13.0 – 25.5) | 20.1 (17.4 – 27.3) | 0.047* | 0.450 |
| VO_2_/kg (ml/kg/min) | 6.90 ± 2.28 | 6.2 ± 3.16 | 0.428 | 4.50 ± 2.12 | 5.2 ± 1.69 | 0.089 | 0.388 |
| VO_2_/kg% | 27.2 (19.8 – 34.5) | 19.8 (18.4 – 26.8) | 0.173 | 16.4 (15.4 – 32.0) | 20.0 (18.5 – 28.7) | 0.093 | 0.880 |
| VCO_2_ (L/min) | 0.25 ± 0.07 | 0.23 ± 0.08 | 0.428 | 0.22 ± 0.06 | 0.213 ± 0.04 | 0.802 | 0364 |
| Respiratory rate (br/min) | 26.56 ± 5.35 | 24.44 ± 6.30 | 0.126 | 27.40 ± 8.10 | 24.78 ± 4.74 | 0.257 | 0.893 |
| Heart rate (b/min) | 106.5 (89.5 - 115.0) | 94 (90.5 - 112.5) | 0.414 | 109.0 (102.5 - 123.5) | 101.0 (93.0 - 106.0) | 0.176 | 0.345 |
| Heart rate% | 60.3 (49.8 – 65.7) | 51.8 (27.1 – 58.3) | 0.500 | 60.6 (58.6 – 67.9) | 57.3 (44.2 – 61.0) | 0.176 | 0.570 |
| SBP (mmHg) | 118 ±7.89 | 120 ± 7.07 | 0.104 | 123 ± 7.89 | 122.5 ± 9.79 | 0.758 | 0.521 |
| DBP (mmHg) | 75.5 ± 9.85 | 76 ± 9.94 | 0.343 | 80.5 ± 9.56 | 79.5 ± 9.27 | 0.443 | 0.426 |
| VO_2_/HR (ml/beat) | 4.29 ± 1.66 | 4.84 ± 2.26 | 0.626 | 2.7 ± 0.95 | 4.14 ± 1.72 | 0.085 | 0.556 |
| VO_2_/HR% | 62.4 (44.7 – 91.2) | 65.6 (43.9 – 95.6) | 0.686 | 59.5 (35.3 – 127.5) | 95.7 (63.6 – 113.7) | 0.018* | 0.465 |
| PETCO_2_ (mmHg) | 23.60 ± 4.43 | 23.7 ± 2.67 | 0.947 | 23.60 ± 2.80 | 24.4 ± 2.12 | 0.235 | 0.524 |
| PETO_2_ (mmHg) | 109.5 (101.0 – 114.0) | 114.0 (113.0 – 115.0) | 0.068 | 118.0 (114.0 – 119.0) | 113.5 (111.0 – 115.0) | 0.160 | 0.436 |
| SpO_2_ (%) | 93.3 ± 2.79 | 94.0 ± 2.36 | 0.066 | 92.3 ± 2.11 | 93.0 ± 2.36 | 0.045***** | 0.355 |
| Breathing Reserve (%) | 83.70 ± 7.56 | 84.5 ± 4.67 | 0.644 | 86.40 ± 4.40 | 86.5 ± 2.95 | 0.923 | 0.268 |
| VD/VT ratio | 0.23 ± 0.08 | 0.29 ± 0.14 | 0.149 | 0.22 ± 0.07 | 0.26 ± 0.13 | 0.128 | 0.674 |

Abbreviations; VE: minute ventilation, br/min: breath/minute, VO_2_: oxygen consumption, VCO_2_: carbon dioxide output, HR: heart rate, VO_2_/HR: oxygen pulse, VD: dead space, VT: tidal volume, RER: respiratory exchange ratio, PETCO_2_: end-tidal carbon dioxide pressure, SpO_2_: oxygen saturation, SBP: systolic blood pressure, DBP: diastolic blood pressure, SD: standard deviation; *significant *p* value < 0.05, ^$^ Comparison between LL and ULB groups after ET, **^#^** Comparison between the same group after ET.

Table S3: Multivariate logistic regression (forward stepwise method) in relation to outcome among studied population

|  | B | S.E. | df | Sig. | OR | CI95% for OR | |
| --- | --- | --- | --- | --- | --- | --- | --- |
|  |  |  |  |  |  | Lower | Upper |
| ET | -2.762 | 1.191 | 1 | 0.020* | 0.063 | 0.006 | 0.652 |
| Constant | -0.182 | 0.606 | 1 | 0.763 | 0.833 |  |  |

ET: exercise training (either lower limb only or upper limbs/lower limbs / breathing), OR: odd ratio, CI: confidence interval. *Significant *p* value < 0.05.

|  |  | Score | df | Sig. |
| --- | --- | --- | --- | --- |
| Variables used in forward method^$^ | Gender | 2.913 | 1 | 0.088 |
|  | Age | 0.545 | 1 | 0.460 |
|  | Smoking status | 0.009 | 1 | 0.923 |
|  | Corticosteroids use | 0.182 | 1 | 0.669 |
| Overall Statistics | | 6.131 | 5 | 0.294 |

^$^ probability of stepwise; entry of variable if *p* < 0.05 otherwise removed from the equation.

**Figures legends**

Fig S1. Aerobic lower limbs exercise training. A. Aerobic treadmill device used, B. Monitor device used during exercise training (for recording of heart rate (HR) and oxygen saturation (SpO_2_)); C. A patient while lower limb aerobic exercise training by treadmill with monitoring of HR and SpO_2_ during the training session.


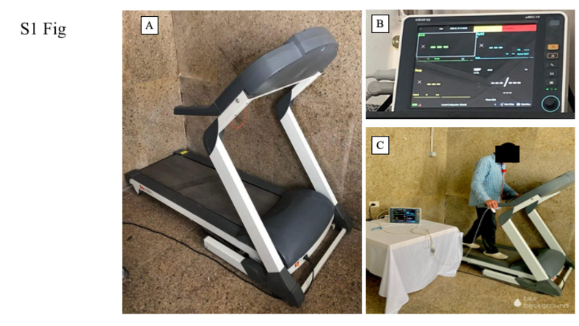


Fig S2: Arm ergometer used for upper limb exercise training.


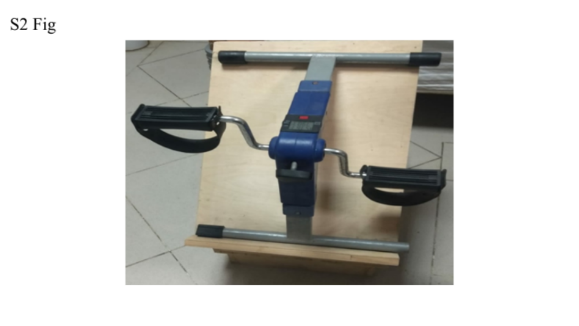

Supplement: S1 File — The file contains methods, S1-S3 Tables and S1, S2 Figs legends. (DOCX) [file pone.0268589.s002.docx]
