# Supplementary material for: Effects of different exercise training programs on the functional performance in fibrosing interstitial lung diseases: A randomized trial
Source: PLoS One. 2022 May 26;17(5):e0268589. doi: 10.1371/journal.pone.0268589 (PMC9135240; doi:10.1371/journal.pone.0268589)
Supplement: S1 Protocol — (PDF) [file pone.0268589.s003.pdf]

EFFECTS OF EXERCISE TRAINING ON RESPIRATORY PERFORMANCE IN PATIENTS WITH FIBROSING INTERSTITIAL LUNG DISEASES

تأثيرات التمرينات الرياضية على أداء الجهاز التنفسي في المرضى المصابين بأمراض الرئة الخلالية الليفية

Protocol of a thesis submitted
to the Faculty of Medicine
University of Alexandria
In partial fulfillment of the
requirements of the degree of
MDPhD in Chest Diseases

By

Hatem Essam Hamed Rizq
MBBCh, Alexandria
Master of Chest Diseases, Alex
Assistant lecturer
Department of Chest Diseases
Faculty of Medicine
University of Alexandria

2019

خطة بحث مقدمة
لكلية الطب
جامعة الإسكندرية
إيفاءً جزئيًاً
لشروط الحصول على درجة
دكتور الفلسفة في الطب في
في الأمراض الصدرية

من

حاتم عصام حامد رزق
بكالوريوس الطب والجراحة، الإسكندرية
ماجستير الأمراض الصدرية، الإسكندرية
مدرس مساعد
قسم الأمراض الصدرية
كلية الطب
جامعة الإسكندرية

٢٠١٩

Approved

Authors

Hatem Essam[1]; MD

Nashwa Hassan Abdel Wahab[1]; MD, PhD

Gihan Younis[2]; MD, PhD

Enas El-sayed[1]; MD, PhD

Hanaa Shafiek[1]; MD, PhD

Affiliations

[1] Chest diseases department, Faculty of Medicine, Alexandria University, Alexandria, Egypt.

[2] Physical Medicine, Rheumatology and Rehabilitation department, Faculty of medicine, Alexandria University, Alexandria, Egypt.

INTRODUCTION

Interstitial lung diseases (ILDs) comprise a heterogeneous group of chronic conditions characterized by lung parenchymal involvement with different degrees of inflammation and fibrosis resulting in impaired gas exchange. [1, 2] Idiopathic pulmonary fibrosis (IPF) is the most common and lethal form.[3]

IPF is, by definition, a progressive fibrosing ILD characterized by decline in lung function and early mortality.[2] Patients with certain other types of chronic fibrosing ILD are also at risk of developing a progressive phenotype. These include idiopathic nonspecific interstitial pneumonia (NSIP),[4] unclassifiable idiopathic interstitial pneumonia,[5] autoimmune ILDs,[6] chronic sarcoidosis,[7] chronic hypersensitivity pneumonitis (HP),[8] and exposure-related diseases such as asbestosis and silicosis.[9]

People with fibrosing ILD frequently experience breathlessness on exertion, which limits their ability to undertake daily activities. [10, 11]. Those with the greatest exercise limitations have the worst quality of life[10], and exercise limitation may be a more robust predictor of prognosis than resting lung function.[12] Abnormal lung mechanics limit ventilatory adaptation to exercise and often lead to a less efficient, rapid, and shallow breathing pattern.[13]

Both chronic and intermittent hypoxia impair skeletal muscle function through a number of mechanisms, including oxidative enzyme inhibition, amino acid efflux, and increased production of reactive nitrogen and oxygen species (ROS).[14]

So, Exercise limitation in fibrosing ILD is multifactorial, with contributions including impairment of gas exchange and pulmonary circulation, ventilatory dysfunction and muscle dysfunction. The latter is an emerging area that might be particularly amenable to amelioration with pulmonary rehabilitation (PR).[12, 15-17]

The 6MWT is the most commonly used test of exercise capacity in PR.[12] The cardiopulmonary exercise testing (CPET) provides detailed information about exercise responses and exercise capacity in ILD. This test is complex and may not be available in all settings.[18]

Available treatments for patients with fibrosing ILD have proved largely ineffective, offering no improvement in survival and demonstrating only limited impact on quality of life.[19]

Pulmonary rehabilitation (PR) programs have been widely assessed and validated in patients with Chronic Obstructive Pulmonary Disease (COPD)[1, 20], for which they have been proved to be effective in reducing respiratory and non-respiratory (i.e. peripheral muscle fatigue) symptoms and improving functional performance status which is consistent with improvement in HRQoL.[1, 21]

Endurance training is an essential component of exercise training for ILD, and most studies have also included resistance training. Endurance training aims to improve aerobic capacity, increase exercise endurance, and improve daily function and physical activity with less breathlessness and fatigue[22]. Resistance training improves local muscle strength and endurance. There are no specific guidelines for prescribing resistance training in PR.[23]

The quadriceps femoris muscle bears the brunt of skeletal muscle affection in ILD. It seems only logical that lower extremity training is the main component of exercise training program. A distinctive pattern of disuse, with greater atrophy and weakness in lower limb muscles compared with the upper limbs and inspiratory muscles is also apparent.[12]

Exercise training could be an effective intervention to improve symptoms, HRQL and functional status in people with ILD.[12]

Despite an increasing level of knowledge regarding the effectiveness of exercise training in patients with ILD, characteristics which determine the success of exercise training in those patients has not been identified clearly.[24]

In summary, existing studies suggest that exercise training is effective across the spectrum of fibrosing ILD and could be offered to all patients who are symptomatic on exertion.

AIM OF WORK

The aim of this study is to compare the effects of aerobic exercises for lower limbs versus both upper, lower limbs, and breathing exercises on the peak exercise measurements using cardiopulmonary exercise testing (CPET), dyspnea and health related quality of life assessment in patients with fibrosing interstitial lung diseases.

PATIENTS

The present study is designed as case-control study that will include 20 patients with fibrosing interstitial lung diseases who present to Alexandria Chest Diseases Department having a stable medical therapy and 10 controls matched in age and sex. The number of the participants is suitable for statistical analysis where the type of test will be chosen according to normal distribution of the data.

The diagnosis of fibrosing ILDs is carried through the radiological features within the high resolution computed tomography of chest, in addition to the restrictive or mixed pattern in spirometric results.[25]

Exclusion criteria:

Items related to contraindications of exercise therapy or assessment tools:[26]

1. Refusal of participation (none consenting).
2. Motor disabilities that hinder exercising.

3. Acute heart failure.
4. Cognitive impairments.
5. Comorbidities (unstable angina, recent myocardial infarction or cerebrovascular accident, active cancer, and a life expectancy below 3 months.

Items related to studied disease:[25]

1. High resolution CT chest with evident ground glass opacities that exclude the chronic fibrosing element of ILD.

2. Evidence of exacerbation of ILD or superadded infection in the previous two weeks.

3. Uncompensated respiratory acidosis on arterial blood gases.

METHODS

Patients who will present to Alexandria Main University Hospitals with fibrosing ILD will give informed consent according to the guidelines of ethics committee, Alexandria Faculty of Medicine (available from www.med.alexu.edu.eg/wp-content/uploads /2012/ 04/ ethics-guide.pdf). Informed consent will be available at (www.med.alexu.edu.eg/wp-content/uploads/2012/04/الموافقه-علي-الخضوع-لبحث-طبي.pdf). The study was approved by local ethical committee (protocol ID: 0201313) on 16-1-2020.

All patients were subjected to baseline assessment as well as outcome assessment.

I- Baseline assessment:

1. Thorough history taking.
2. Complete physical examination

3. Routine laboratory investigations.
4. Investigations: high resolution computed tomography of chest and spirometry to confirm the diagnosis of fibrosing ILD.
5. The following measurements were recorded for all patients at baseline:
    - Dyspnea assessment (by mMRC scale).[27]
    - Pulmonary function tests.[28]
    - Measurement of 6 Minute Walk distance (6MWT).[29]
    - Exercise measurements using cardiopulmonary exercise testing.[30]
    - Quality of life assessment (via St. George's Respiratory Questionnaire "SGRQ").[31]

II- <u>The exercise training (ET) program</u>:

The ET program will consist of aerobic exercises for lower and upper limbs and breathing exercises that will be done in 3 sessions per week for 6 weeks with a duration of 15 minutes. The type of exercise used will be tailored according to each patient.

All patients will be randomly divided into 2 groups.

Group I: patients in this group will perform aerobic exercise training for lower limbs (n= 10).

Group II: Patients in this group will perform upper limb and breathing exercises in addition to the lower limb training (n= 10).

Group III: Patients in this group will be considered as control (no exercise training will be provided) and general care will be offered (n= 10).

III- <u>Outcome assessment:</u>

Assessments will be done by the end of 6 weeks of the program using the following parameters:

- The modified Medical Research Council dyspnea scale.
- Spirometry.
- Measurement of 6 Minute Walk distance (6MWT).
- Physiological measurements during exercise using cardiopulmonary exercise testing.
- The St George's respiratory questionnaire as a measure of health-related QoL.

ETHICS OF RESEARCH

Research on human or human products:

- [x] Prospective study: Informed consent will be taken from patients. In case of incompetent patients the informed consent will be taken from the guardians.
- [ ] Retrospective study: Confidentiality of records will be considered
- [ ] DNA / genomic material: Informed consent for DNA / genomic test and for research will be taken from patients. No further tests will be carried out except with further approval of committee and patients. If the samples will travel outside Egypt the researcher will be responsible for transportation and security approval.
- [ ] All drugs used in the research are approved by the Egyptian Ministry of Health

---

Research on animal: None

- [ ] The animal species are appropriate for the test.
- [ ] After test, if the animal will suffer, it will be euthanized and properly disposed.
- [ ] After operation, it will have a proper postoperative care.

RESULTS

The results obtained from this study will be analyzed using appropriate statistical methods. Normal distribution of the data will be evaluated. For comparison between two groups, Mann-Whitney or t- test will be used according to the normal distribution of the data. For comparison between 3 groups, C*hi-square test,* one-way ANOVA test and Kruskal-Wallis will be used. Paired *t*-test and Wilcoxon signed rank will be used for the comparison between the baseline and the follow up of the intervention groups.

The results obtained from this study will be discussed in view of achievement of the aim of the work and will be compared to all available published data in the same field of the research.

REFERENCES

1. Tonelli R, Cocconcelli E, Lanini B, Romagnoli I, Florini F, Castaniere I, et al. Effectiveness of pulmonary rehabilitation in patients with interstitial lung disease of different etiology: a multicenter prospective study. BMC Pulmonary Medicine. 2017;17(1):130.
2. Raghu G, Collard HR, Egan JJ, Martinez FJ, Behr J, Brown KK, et al. An official ATS/ERS/JRS/ALAT statement: idiopathic pulmonary fibrosis: evidence-based guidelines for diagnosis and management. Am J Respir Crit Care Med. 2011;183(6):788-824.
3. Travis WD, Costabel U, Hansell DM, King Jr TE, Lynch DA, Nicholson AG, et al. An official American Thoracic Society/European Respiratory Society statement: update of the international multidisciplinary classification of the idiopathic interstitial pneumonias. Am J Respir Crit Care Med. 2013;188(6):733-48.
4. Belloli EA, Beckford R, Hadley R, Flaherty KR. Idiopathic non-specific interstitial pneumonia. Respirology. 2016;21(2):259-68.
5. Hyldgaard C, Bendstrup E, Wells AU, Hilberg O. Unclassifiable interstitial lung diseases: clinical characteristics and survival. Respirology. 2017;22(3):494-500.
6. Fischer A, Chartrand S. Assessment and management of connective tissue disease-associated interstitial lung disease. Sarcoidosis Vasc Diffuse Lung Dis. 2015;32(1):2-21.
7. Patterson KC, Strek ME. Pulmonary fibrosis in sarcoidosis. Clinical features and outcomes. Ann Am Thorac Soc. 2013;10(4):362-70.
8. Pérez ERF, Swigris JJ, Forssén AV, Tourin O, Solomon JJ, Huie TJ, et al. Identifying an inciting antigen is associated with improved survival in patients with chronic hypersensitivity pneumonitis. Chest. 2013;144(5):1644-51.

9. Khalil N, Churg A, Muller N, O'Connor R. Environmental, inhaled and ingested causes of pulmonary fibrosis. Toxicol Pathol. 2007;35(1):86-96.

10. Collard HR, Talmadge E. King J, Bartelson BB, Vourlekis JS, Schwarz MI, et al. Changes in Clinical and Physiologic Variables Predict Survival in Idiopathic Pulmonary Fibrosis. Am J Respir Crit Care Med. 2003;168(5):538-42.

11. Flaherty KR, Andrei A-C, Murray S, Fraley C, Colby TV, Travis WD, et al. Idiopathic Pulmonary Fibrosis. Am J Respir Crit Care Med. 2006;174(7):803-9.

12. Nakazawa A, Cox NS, Holland AE. Current best practice in rehabilitation in interstitial lung disease. Ther Adv Respir Dis. 2017;11(2):115-28.

13. Jones NL, Killian KJ. Exercise limitation in health and disease. N Engl J Med. 2000;343(9):632-41.

14. Clanton TL. Hypoxia-induced reactive oxygen species formation in skeletal muscle. J Appl Physiol (1985). 2007;102(6):2379-88.

15. Kozu R, Senjyu H, Jenkins SC, Mukae H, Sakamoto N, Kohno S. Differences in response to pulmonary rehabilitation in idiopathic pulmonary fibrosis and chronic obstructive pulmonary disease. Respiration. 2011;81(3):196-205.

16. Nishiyama O, Taniguchi H, Kondoh Y, Kimura T, Ogawa T, Watanabe F, et al. Quadriceps weakness is related to exercise capacity in idiopathic pulmonary fibrosis. Chest. 2005;127(6):2028-33.

17. Spruit MA, Thomeer MJ, Gosselink R, Troosters T, Kasran A, Debrock AJT, et al. Skeletal muscle weakness in patients with sarcoidosis and its relationship with exercise intolerance and reduced health status. Thorax. 2005;60(1):32-8.

18. Albouaini K, Egred M, Alahmar A, Wright DJ. Cardiopulmonary exercise testing and its application. Postgraduate Medical Journal. 2007;83(985):675-82.

19. Holland AE, Spruit MA, Troosters T, Puhan MA, Pepin V, Saey D, et al. An official European Respiratory Society/American Thoracic Society technical

standard: field walking tests in chronic respiratory disease. Eur Respir J. 2014;44(6):1428-46.

20. Vestbo J, Hurd SS, Rodriguez-Roisin R. The 2011 revision of the global strategy for the diagnosis, management and prevention of COPD (GOLD) – why and what? Clin Respir J. 2012;6(4):208-14.

21. Nici L, Donner C, Wouters E, Zuwallack R, Ambrosino N, Bourbeau J, et al. American Thoracic Society/European Respiratory Society Statement on Pulmonary Rehabilitation. Am J Respir Crit Care Med. 2006;173(12):1390-413.

22. Cadore EL, Casas-Herrero A, Zambom-Ferraresi F, Idoate F, Millor N, Gómez M, et al. Multicomponent exercises including muscle power training enhance muscle mass, power output, and functional outcomes in institutionalized frail nonagenarians. AGE. 2014;36(2):773-85.

23. Kraemer WJ, Ratamess NA, French DN. Resistance training for health and performance. Curr Sports Med Rep. 2002;1(3):165-71.

24. Kaymaz D, Ergün P, Candemir I, Utku E, Demir N, Sengül F, et al. Pulmonary rehabilitation in interstitial lung diseases. Tuberk Toraks. 2013;61(4):295-302.

25. Cottin V, Hirani NA, Hotchkin DL, Nambiar AM, Ogura T, Otaola M, et al. Presentation, diagnosis and clinical course of the spectrum of progressive-fibrosing interstitial lung diseases. Eur Respir Rev. 2018;27(150).

26. Society AT. ATS/ACCP statement on cardiopulmonary exercise testing. Am J Respir Crit Care Med. 2003;167(2):211.

27. Hsu K-Y, Lin J-R, Lin M-S, Chen W, Chen Y-J, Yan Y-H. The modified Medical Research Council dyspnoea scale is a good indicator of health-related quality of life in patients with chronic obstructive pulmonary disease. Singapore Med J. 2013;54(6):321-7.

28. Enright PL, Lebowitz MD, Cockroft DW. Physiologic measures: pulmonary function tests. Am J Respir Crit Care Med. 2012.

29. Enright PL. The six-minute walk test. Respir Care. 2003;48(8):783-5.
30. Radtke T, Crook S, Kaltsakas G, Louvaris Z, Berton D, Urquhart DS, et al. ERS statement on standardisation of cardiopulmonary exercise testing in chronic lung diseases. Eur Respir Rev. 2019;28(154).
31. Jones PW, Quirk FH, Baveystock CM, Littlejohns P. A self-complete measure of health status for chronic airflow limitation. Am Rev Respir Dis. 1992;145(6):1321-7.
