# Supplementary material for: Effects of different exercise training programs on the functional performance in fibrosing interstitial lung diseases: A randomized trial
Source: PLoS One. 2022 May 26;17(5):e0268589. doi: 10.1371/journal.pone.0268589 (PMC9135240; doi:10.1371/journal.pone.0268589)
Supplement: Attachment — Submitted filename: Plos One f-ILD (2).docx [file pone.0268589.s004.docx]

Dear editor,

Thank you for giving me into the charge to review this article, I am very honoured.

Although the number of cases in this study is small, exercise test is an important issue for management of interstitial lung diseases and treatment options are limited. Therefore, I think that the study contributes to the literature. So this article is acceptable after revisions.

My comments are written below:

- **Abstract:**

In my opinion, unit forVO_2_should be added.

- **In methods section :**

I thought it would be better if images about aerobic exercise tests were used ( the monitor of the device, etc.)

DLCO is crucial in interstitial lung diseases. Is there any particular reason for not mentioning about it?

The literature or information about mMRC dispnea scale should be added.

- **InStatistical analysis:**

**Line 153**‘All the data were expressed as median and interquartile range (IQR) or mean . standard deviation(SD) according to the normal distribution of continuous data’.

This sentence hasa missing part. “Median and interquartile rangefor non-normal distribution“should be added.

- **In the Results:**

**Table 1** has both weight and BMI values. In my opinion, emphasizing “weight” as a separate entity is not necessary. Also, “Y/N” statements for comorbidities have only Y statement in the table. So, “N” statement should be removed.

**In Table 1**CPEE is considered to be a phenotype of IPF, so it can be merged.

**182**

“The VO2, VO2% predicted,VO2/kg and oxygen pulse (VO2/HR) at baseline were significantly lower among ULB groups compared to LL group and control group (p < 0.05, table S1 online supplement)”Could you please check this sentence? Only VO2/HR is statistically significant according to Table 1.

**184-185**“At maximal exercise, SpO2 of the control group was significantly lower when…”Isn’t the control group selected from the ones do not exercise??

**186-187** ‘while the oxygen pulse was significantly lower among ULB group

versus both LL and control groups (p= 0.045, table 2)’ I didn’t see these results in table 2.

**210-211**‘VO2, VO2% predicted, VO2/HR% predicted and SpO2 at baseline significantly improved after ET training among ULB group (p < 0.05, table S2, online supplement) but not after LL only training program (p > 0.05, table S2, online supplement)’.

In table 4, VO2 and VO2/HR%, are not statistically significant for ULB group.

**212-213**

‘After training, resting HR in both groups were reduced and VT increased but not at a significant level (p > 0.05, table S2, online supplement’

Could you please check this sentence? VT is decreased significantly in table 4. HR results for both groups are different.

**215**- **‘**peak VO2 after ET in both groups (LL only and ULB) (p < 0.05, table 4)’ Could you pleasecheck this sentence? This statement is not statistically significant according to table 4.
